# Cervicovaginal dysbiosis and microenvironment disruption are associated with cervical carcinogenesis

**DOI:** 10.1128/spectrum.02804-24

**Published:** 2026-04-16

**Authors:** Meng Cui, Mingxuan Zhang, Jianjun Zhang, Rui Mao, Ling Ding, Zhilian Wang, Li Song, Yuanjing Lyu, Huimin Li, Kailu Zhao, Jintao Wang

**Affiliations:** 1 Department of Epidemiology, School of Public Health, Shanxi Medical University 572534, Taiyuan, China; 2 Department of Community Geriatric Nursing, School of Nursing, Inner Mongolia Medical University, Hohhot, China; 3 Shanghai Pudong New Area Center for Disease Control and Prevention (Shanghai Pudong New Area Health Supervision Institute) 12478, Shanghai, China; 4 Fudan University Pudong Institute of Preventive Medicine, Shanghai, China; 5 Department of Epidemiology, Fairbanks School of Public Health, Indiana University 1772, Bloomington, Indiana, USA; 6 Questrom School of Business, Boston University 1846, Boston, Massachusetts, USA; 7 Department of Obstetrics and Gynecology, Second Hospital of Shanxi Medical University 74761, Taiyuan, China; Hubei University of Medicine, Shiyan, China; All Indian Institute of Medical Sciences, Rishikesh, Uttarakhand, India; Joseph Sarwuan Tarka University Makurdi, Makurdi, Benue, Nigeria

**Keywords:** cervicovaginal microbiota, vaginal microenvironment, cervical cancerization, 16S rRNA gene sequencing

## Abstract

Cervical cancer poses a serious threat to women’s health. Emerging evidence indicates that changes in the cervicovaginal microbiota (CVM) and vaginal microenvironment may play a crucial role in cervical carcinogenesis. In this study, we examined CVM and vaginal microenvironment profiles in 510 participants with varying stages of cervical lesions using 16S rRNA sequencing, vaginal pH and H_2_O_2_ measurements, and cleanness assessment. A co-occurrence network and generalized multifactor dimensionality reduction model were employed to analyze the correlation and interaction between microbial taxa and microenvironment profile. Our results demonstrated that CVM disorder and abnormal vaginal microenvironment were associated with cervical lesions. As cervical cancerization progressed, the abundance of *Lactobacillus* gradually decreased with thriving anaerobe levels in the deteriorating vaginal microenvironment. This interaction was characterized by high vaginal pH, a non-*Lactobacillus*-dominant microbiota, and high CVM diversity (Shannon index ≥ 0.81). Co-occurrence network analysis revealed that abnormal pH and H_2_O_2_ levels correlated with *Lactobacillus* depletion and had a positively co-occurring relationship with anaerobes in precancerous cervical lesions. CVM function significantly varied with changes in the vaginal microenvironment across different stages of cervical lesions. Our findings suggest that CVM dysbiosis, particularly when combined with an abnormal vaginal microenvironment, may promote the progression of cervical lesions. This highlights the importance of addressing vaginal microecology to prevent cervical carcinogenesis.

**IMPORTANCE:**

Cervicovaginal microbiota (CVM) and vaginal microenvironment in human papillomavirus infection are associated with the progression of cervical carcinogenesis. CVM dysbiosis, particularly the decrease in *Lactobacillus* and the increase in anaerobes, is crucial in promoting the malignant progression of cervical lesions. As cervical carcinogenesis progresses, the relative abundance of anaerobes increases in the deteriorating vaginal microenvironment. We systematically analyzed the relationship between CVM and the vaginal microenvironment in cervical lesions, aiming to reveal the contribution of CVM with specific vaginal microenvironmental features and its functional changes in the progression of cervical carcinogenesis. Our results may provide new insights into the etiology and pathogenesis of cervical cancer from the perspective of vaginal microecology.

## INTRODUCTION

Cervical cancer represents the fourth most common malignancy among females worldwide (1). Persistent infection with high-risk human papillomavirus (HPV) is a key etiological factor driving cervical carcinogenesis (2). However, not all women infected with HPV develop cervical lesions, suggesting that additional factors may influence the progression of cervical carcinogenesis (3).

The vagina connects the cervix to the external environment and is colonized by a diverse community of microorganisms within its mucous membranes (4). The acidic environment of the vagina is maintained by the microbiota of the cervix and vagina, which is dominated by *Lactobacillus*. *Lactobacillus* species produce lactic acid, H_2_O_2_, bacteriocin, and other antimicrobial compounds that protect against infections and maintain the acidic environment (pH ≤ 4.5) (5). A cervicovaginal microbiota (CVM) dominated by *Lactobacillus* serves as an effective first-line defense against invading pathogens, thereby preserving vaginal health (6). Disruption of CVM can lead to the overgrowth of pathogens associated with several gynecological diseases (7). Multiple studies have consistently shown that reduced dominance of *Lactobacillus* and an increased abundance of pathogenic bacteria, such as *Gardnerella* and *Sneathia*, are often observed in women with cervical cancer (8). Thus, CVM dysbiosis may play a crucial role in cervical carcinogenesis (9). It is important to note that microorganisms coexist and interact with each other, and these complex interactions can mutually influence their functional activities (10). However, the biological interactions and the functional changes of CVM during the occurrence and development of cervical cancer are not yet fully understood. Specifically, it is still unknown whether CVM dysbiosis and its associated functional changes, particularly when combined with an abnormal vaginal microenvironment, contribute to cervical carcinogenesis. Furthermore, it remains unclear if these factors could serve as valuable markers for the development of cervical dysplasia and the progression to invasive carcinoma.

The balance of vaginal microecology is closely related to the vaginal microenvironment. Indicators such as vaginal pH, H_2_O_2_ levels, and cleanness reflect the vaginal microenvironment profile, including *Lactobacillus* function, microbial metabolites, and inflammatory reaction, and are practical indices for clinical gynecological check-ups (11). An imbalanced vaginal microenvironment is associated with cervical carcinogenesis (12). Our previous work revealed that vaginal microenvironment disorder could increase the risk of cervical intraepithelial neoplasia (CIN) (13). However, the association between CVM dysbiosis and vaginal microenvironment disorder, as well as their interaction in cervical carcinogenesis, remains unclear.

Herein, we aimed to highlight the role of CVM and vaginal microenvironment in HPV infection in the progression of cervical carcinogenesis. Our results may provide new insights into the etiology and pathogenesis of cervical cancer from the perspective of vaginal microecology.

## MATERIALS AND METHODS

### Study population

A total of 510 women were enrolled in this study, including 290 women with normal cervical histology (NC), 204 patients with CIN, and 16 patients with cervical squamous cell carcinoma (SCC) diagnosed by pathology. The participants were recruited from the Obstetrics and Gynecology Department of Shanxi Medical University Second Hospital, Shanxi, China, from January to June 2018. The inclusion criteria were Han ethnicity, residence in Shanxi for at least 5 years, and a history of sexual activity for more than 2 years. The exclusion criteria were as follows: a history of hysterectomy or cervical conization treatment, the presence of other malignant tumors, an immune system or mental illness, and pregnancy or lactation. Among them, 73 women (22 with NC, 41 with CIN, and 10 with SCC) who had not used antibiotics, vaginal medication, or vaginal douching within 3 days prior to the study and were matched for age and residence (same city/county) across different groups were selected for 16S rRNA gene sequencing.

### Data and sample collection

Information on demographic characteristics and factors related to cervical lesions was collected using a structured questionnaire. Samples of vaginal secretion were obtained using a sterile cotton swab from the posterior vaginal fornix and placed into 3 mL of sterile saline. Vaginal pH, H_2_O_2_ levels, and cleanness of the secretion specimens were measured within 15 min. Additionally, cervical exfoliative cells were collected using a cytobrush for CVM detection via 16S rRNA sequencing.

### 16S rRNA sequencing

CVM DNA was extracted from the samples of vaginal secretion using the E.Z.N.A. Soil DNA Kit (Omega Biotek, Norcross, GA, USA). The integrity of the DNA was verified by agarose gel electrophoresis. The V3–V4 regions of the 16S rRNA gene were amplified by PCR using the primers 341F (CCTACGGGNGGCWGCAG) and 805R (GACTACHVGGGTATCTAATCC). Following DNA purification, recovery, and accurate quantitative mixing of PCR products, 16S rRNA sequencing was performed on an Illumina MiSeq 2 × 300 bp platform (Illumina, San Diego, CA, USA) by Sangon Biotech Co., Ltd. (Shanghai, China).

### Measurements of vaginal microenvironment profile

In line with previous descriptions (13), the collected samples of vaginal secretion were immediately tested for vaginal pH, H_2_O_2_, and cleanness. Briefly, vaginal pH was measured using color strips with a pH range of 3.8–5.4, where a pH ≤ 4.5 was considered normal, while a pH > 4.5 was considered abnormal. Vaginal H_2_O_2_ levels were measured using the Aerobic Vaginitis (AV)/Bacterial Vaginal Disease (BV) Five Joint Test Kit (Beijing ZhongSheng JinYu Diagnosis Technology Co., Ltd., Beijing, China), with negative results indicating normal H_2_O_2_ levels and positive results indicating abnormal levels. Vaginal cleanness was assessed by observing the secretion sample fluid smear under a high-powered microscope, with grading from I to IV. In line with the National Clinical Laboratory Practice Guideline (14), grades I and II were defined as normal, whereas grades III and IV were considered abnormal.

### Bioinformatics analysis

The Quantitative Insights Into Microbial Ecology software (QIIME, version 1.8.0; http://qiime.org/) was used to extract high-quality sequences from MiSeq sequencing. Sequences and chimera were identified and removed using Usearch (version 5.2.236; https://www.drive5.com/usearch/), and effective sequences were categorized into operational taxonomic units (97% identity threshold), classified using the RDP Naïve Bayesian Classifier (version 2.12; https://sourceforge.net/projects/rdp-classifier/).

The Shannon index was evaluated using Mothur (version 1.30.1; http://www.mothur.org/) to measure alpha diversity. Principal coordinates analysis (PCoA) was used to analyze beta diversity with the R vegan package (version 2.0-10). The linear discriminant analysis (LDA) effect size (LEfSe; http://huttenhower.sph.harvard.edu/galaxy/) method was used to identify biomarkers, with an LDA score >3.0 and *P* < 0.05 indicating significance (15).

Based on Spearman’s rank correlation results (*ρ* > 0.3 and *P* < 0.05), elements from CVM, vaginal pH, H_2_O_2_, and cleanness were represented as the nodes in the co-occurrence network. Significant correlations between nodes were shown as edges in the network plots (https://www.bioincloud.tech/).

Phylogenetic Investigation of Communities by Reconstruction of Unobserved States (PICRUSt, version 1.0.0; http://picrust.github.io/picrust/) was used to predict microbial functions based on the Kyoto Encyclopedia of Genes and Genomes (KEGG; https://www.genome.jp/kegg/) pathways database (16). Differences were analyzed using STAMP (version 2.1.3; https://kiwi.cs.dal.ca/Software/STAMP) with the Welch’s two-sided two-group *t*-test to compare the mean proportion of the KEGG level 3 pathways among the groups.

### Statistical analysis

Data analysis was performed using the Statistical Package for the Social Sciences (SPSS, version 24.0). The chi-square and trend chi-square tests were used for count data, and the Student-Newman-Keuls test was used to analyze differences between any two groups. Non-normally distributed data were analyzed using the Wilcoxon rank-sum test and Kruskal-Wallis *H* test. The multinomial logistic regression model estimated odds ratios (ORs) and 95% confidence intervals (CIs). The interaction between CVM and the vaginal microenvironment was analyzed using generalized multifactor dimensionality reduction (GMDR). Statistical significance was set at *α* = 0.05.

## RESULTS

### Demographic characteristics and factors related to cervical carcinogenesis in the participants

Potential risk factors of cervical carcinogenesis among the 290 participants with NC, 204 participants with CIN, and 16 participants with SCC are presented in Table 1. Age, passive smoking, frequency of bathing, frequency of pubic cleaning, frequency of washing underwear, parity, history of vaginitis, age of the first sexual intercourse, menopause, and HPV infection significantly differed among the three groups (*P* < 0.05).

**TABLE 1:** Potential risk factors related to cervical carcinogenesis^*a*^

Variables	NC (%)	CIN (%)	SCC (%)	*χ* ^2^	*P*
Age (years)				14.003	0.006
<35	54 (18.6)	48 (23.5)	1 (6.2)		
35–54	168 (57.9)	117 (57.4)	5 (31.3)		
≥55	68 (23.5)	39 (19.1)	10 (62.5)		
Passive smoking				7.832	0.02
Yes	174 (60.0)	144 (70.6)	13 (81.2)		
No	116 (40.0)	60 (29.4)	3 (18.8)		
Frequency of bathing				15.181	0.004
>1 time/week	122 (42.1)	78 (38.2)	1 (6.3)		
1 time/week to 1 time/month	154 (53.1)	120 (58.8)	12 (75.0)		
<1 time/month	14 (4.8)	6 (3.0)	3 (18.7)		
Frequency of pubic cleaning				9.582	0.048
>1 time/week	247 (85.2)	162 (79.4)	10 (62.5)		
1 time/week to 1 time/month	34 (11.7)	36 (17.6)	4 (25.0)		
<1 time/month	9 (3.1)	6 (3.0)	2 (12.5)		
Frequency of washing underwear				20.818	<0.001
<2 times/week	192 (66.2)	134 (65.7)	3 (18.8)		
2 times/week to 3 times/week	75 (25.9)	58 (28.4)	8 (50.0)		
>3 times/week	23 (7.9)	12 (5.9)	5 (31.2)		
History of vaginitis				6.8	0.033
Yes	130 (44.8)	83 (40.7)	2 (12.5)		
No	160 (55.2)	121 (59.3)	14 (87.5)		
Age at the first intercourse				11.238	0.024
<20 years	67 (23.1)	50 (24.5)	8 (50.0)		
20–25 years	183 (63.1)	137 (67.2)	5 (31.3)		
>25 years	40 (13.8)	17 (8.3)	3 (18.8)		
Numbers of birth				9.174	0.011
<3	229 (79.0)	167 (81.9)	8 (50.0)		
≥3	61 (21.0)	37 (18.1)	8 (50.0)		
Menopause				25.215	<0.001
Yes	92 (31.7)	47 (23.0)	13 (81.3)		
No	198 (68.3)	157 (77.0)	3 (18.7)		
HPV infection				11.872	0.003
HPV+	257 (88.6)	197 (96.6)	16 (100.0)		
HPV−	33 (11.4)	7 (3.4)	0 (0.0)		
HPV infections type				12.152*	0.044
Single infection: HR-HPV (HPV16, 18, 31, 33, 35, 39, 45, 51, 52, 53, 56, 58, 59, 66, 68)	187 (64.5)	141 (69.1)	12 (75.0)		
Single infection: LR-HPV (HPV6, 11, 42, 43, 44, CP8304)	7 (2.4)	4 (2.0)	0 (0.0)		
Multiple infections	63 (21.7)	52 (25.5)	4 (25.0)		
Negative	33 (11.4)	7 (3.4)	0 (0.0)		
Education attainment				8.973	0.055
Primary school or below	43 (14.8)	35 (18.1)	6 (37.5)		
Middle school	153 (52.8)	99 (48.5)	9 (56.3)		
University or above	94 (32.4)	68 (33.4)	1 (6.2)		
Marital status				4.76	0.093
Non-divorced	254 (87.6)	178 (87.3)	11 (68.8)		
Divorced or widowed	36 (12.4)	26 (12.7)	5 (31.2)		
Occupation				4.222	0.377
Medical, scientific, educational staff	97 (33.5)	67 (32.8)	4 (25.0)		
Worker	27 (9.3)	10 (4.9)	1 (6.3)		
Other	166 (57.2)	127 (62.3)	11 (68.7)		

^*a*^ “*” indicates statisticallly significant difference (*P *< 0.05).

### CVM dysbiosis is associated with cervical carcinogenesis

**Fig 1:**
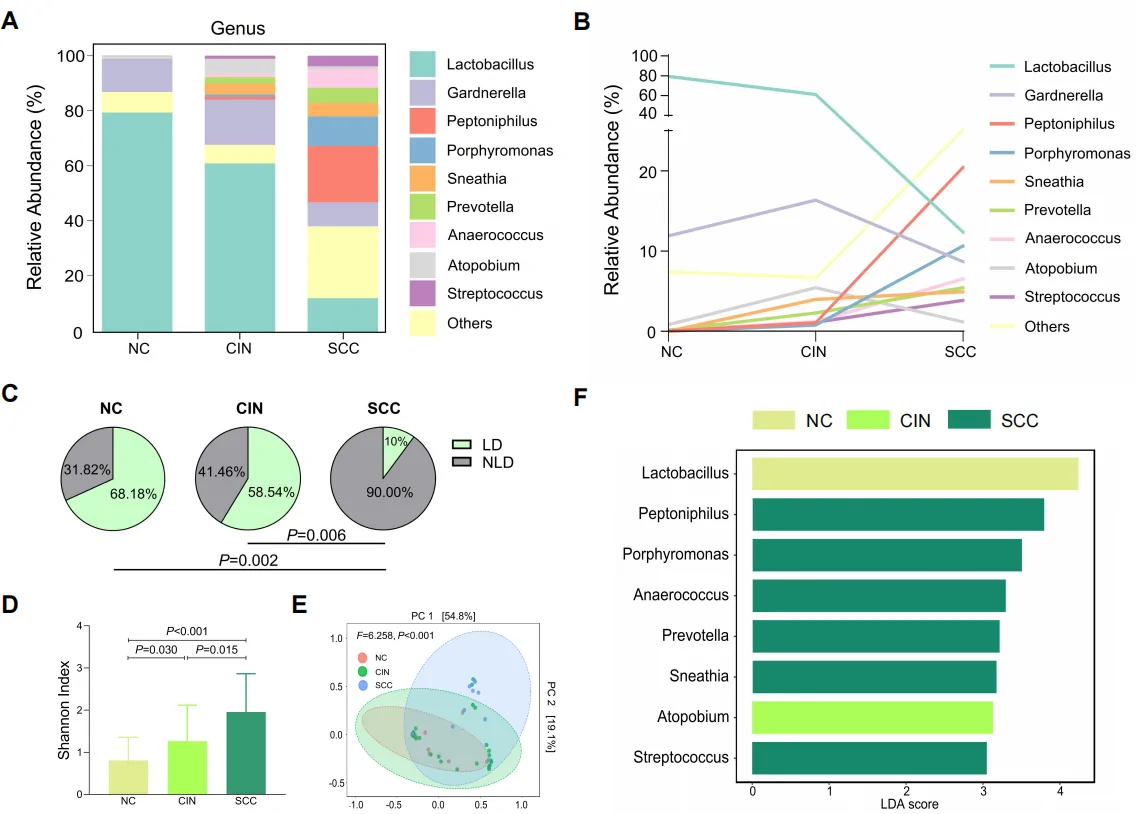
Comparison of the cervicovaginal microbiota structure in different stages of cervical carcinogenesis. (**A**) Cervicovaginal microbiota composition in different groups. (**B**) The trend of microflora changes with the progression of cervical lesions. (**C**) Change in *Lactobacillus* dominance with the progression of cervical lesions. LD, *Lactobacillus* dominance (*Lactobacillus* relative abundance ≥80%); NLD, non-*Lactobacillus* dominance (*Lactobacillus* relative abundance <80%). (**D**) Alpha diversity index of the cervicovaginal microbiota between the groups; alpha diversity is represented as the Shannon index, which shows a positive correlation with the diversity of the cervicovaginal microbiota. *, a statistically significant difference (*P* < 0.05). (**E**) PCoA of the cervicovaginal microbiota among the groups. The cervicovaginal microbiota of the patients with SCC formed a separate cluster from the NC or CIN patients on principal component 1 (PC1) and PC2, which explained 73.9% of the variation in the data set (permutational multivariate analysis of variance: *F* = 6.258, *P* < 0.001). (**F**) LEfSe analysis identified the cervicovaginal microbiota’s differentially abundant taxa among the groups with an LDA score >3.0 and *P* < 0.05 indicating significance. The length of the histogram represents the impact of the significant genera for different cervical lesions.

*Lactobacillus* was the most enriched component in the NC (79.42%) and CIN (61.05%) groups, whereas *Peptoniphilus* (20.42%) was notably abundant in the SCC group (Fig. 1A). As the severity of cervical lesions increased, *Lactobacillus* levels decreased, whereas anaerobes, particularly *Peptoniphilus*, *Sneathia*, *Prevotella*, *Porphyromonas*, *Anaerococcus*, and *Streptococcus*, proliferated (Fig. 1B; Fig. S1). The rates of non-*Lactobacillus* dominance (NLD, *Lactobacillus* relative abundance <80%) increased with the progression of cervical carcinogenesis (*χ*^2^_trend_ = 7.31, *P*_trend_ = 0.007, Fig. 1C). Alpha diversity also increased with the progression of cervical lesions (*F* = 7.570, *P* = 0.001, Fig. 1D), and beta diversity showed significant differences among the groups (*P* < 0.001, Fig. 1E). *Atopobium* was enriched in the CIN group, whereas other anaerobes were significantly over-represented in the SCC group (Fig. 1F).

### Vaginal microenvironment profile in cervical carcinogenesis

A higher percentage of women in the CIN group had abnormal vaginal pH and H_2_O_2_ levels than in the NC group, and these levels increased in line with the severity of cervical cancer. Abnormal vaginal pH (aOR = 2.03, 95% CI: 1.21–3.41) and H_2_O_2_ (aOR = 2.08, 95% CI: 1.30–3.34) were associated with the development of CIN. No similar results were observed for vaginal cleanness. Overall, an abnormal vaginal microenvironment was linked to the progression of cervical lesions, especially an increased risk for CIN (Table 2).

**TABLE 2:** Association between vaginal microenvironment and cervical lesions^*a*^

Factor	Group	*N*	Abnormal (%)	*χ* ^2^	*P*	OR (95% CI)	aOR (95% CI)^*b*^
pH	NC	290	226 (77.9)			1.00 (reference)	1.00 (reference)
	CIN	204	175 (85.8)	4.83	0.028	1.71 (1.06–2.77)	2.03 (1.21–3.41)
	SCC	16	15 (93.8)	1.42	0.233	4.25 (0.55–32.77)	1.44 (0.13–15.97)
*χ*^2^ *=* 6.54, *P* = 0.038; *χ*^2^_trend_ *=* 6.53, *P* = 0.011							
H_2_O_2_	NC	290	208 (71.7)			1.00 (reference)	1.00 (reference)
	CIN	204	165 (80.9)	5.43	0.020	1.67 (1.08–2.57)	2.08 (1.30–3.34)
	SCC	16	15 (93.8)	2.69	0.101	5.91 (0.77–45.49)	2.53 (0.25–25.81)
*χ*^2^ *=* 8.35, *P* = 0.015; *χ*^2^_trend_ *=* 8.25, *P* = 0.004							
Cleanness	NC	290	61 (21.0)			1.00 (reference)	1.00 (reference)
	CIN	204	43 (21.1)	<0.001	0.991	1.00 (0.65–1.56)	1.12 (0.70–1.78)
	SCC	16	5 (31.3)	0.43	0.512	1.71 (0.57–5.10)	1.39 (0.38–5.15)
*χ*^2^ *=* 0.96, *P* = 0.619; *χ*^2^_trend_ *=* 0.24, *P* = 0.620							

^*a*^ aOR, adjusted odds ratio; CI, confidence interval; CIN, cervical intraepithelial neoplasia; NC, cervical histology; OR, odds ratio; SCC, cervical squamous cell carcinoma.

^*b*^ aOR represents the OR adjusted for age, passive smoking, frequency of bathing, frequency of pubic cleaning, frequency of washing underwear, history of vaginitis, age at first intercourse, parity, menopause, and HPV infection type.

### CVM disturbances under an abnormal microenvironment promote malignant progression of cervical lesions

**Fig 2:**
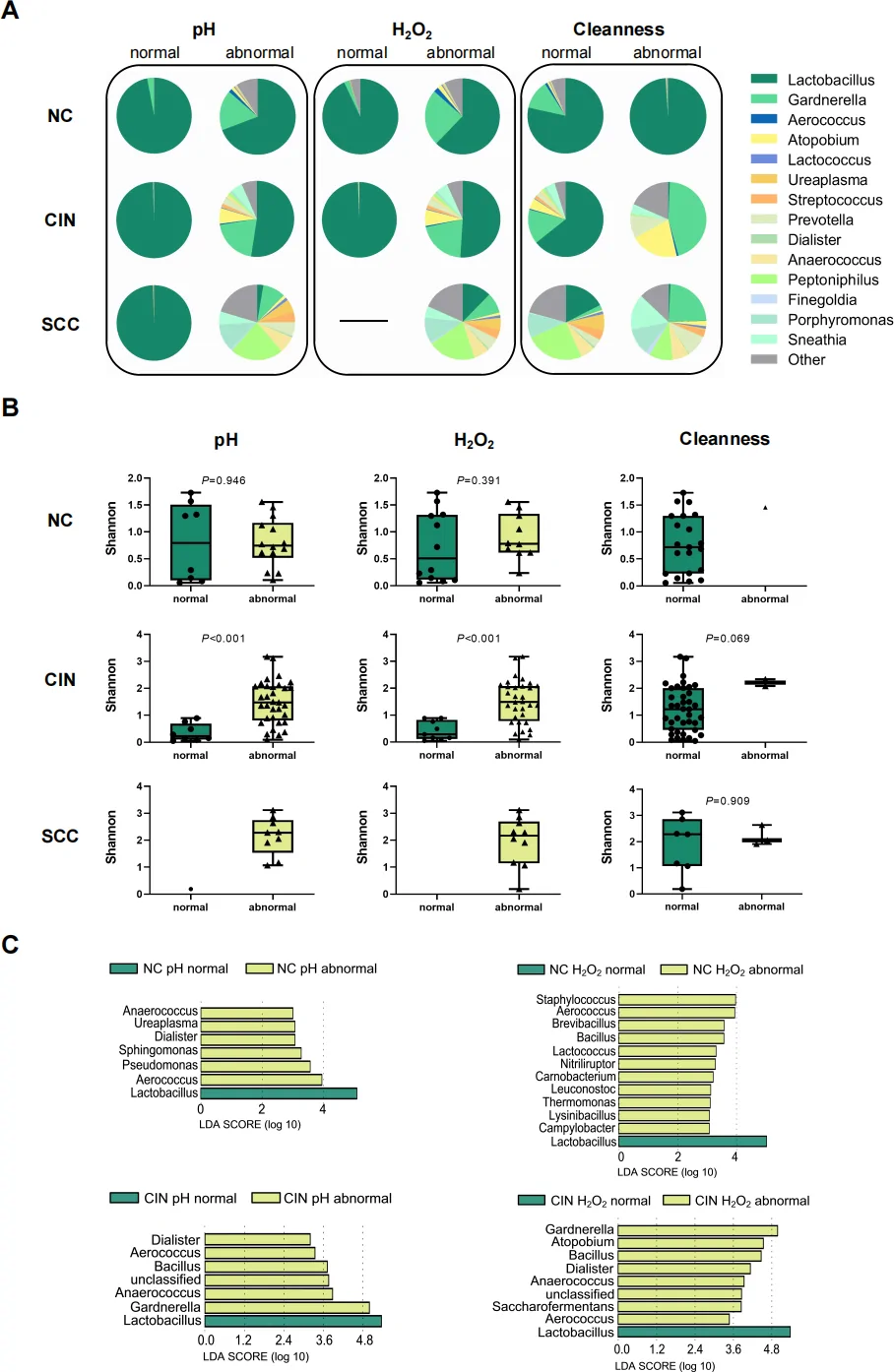
Characteristics of cervicovaginal microbiota in different stages of cervical carcinogenesis in relation to vaginal microenvironment. (**A**) Cervicovaginal microbiota distribution in different cervical lesions in relation to vaginal microenvironment status. The abnormal rate of H_2_O_2_ was 100% in the SCC group. (**B**) Cervicovaginal microbiota diversity in different cervical lesions in relation to vaginal microenvironment status. All SCC patients had abnormal vaginal H_2_O_2_. *P* values were calculated using the Wilcoxon rank-sum test. (**C**) LEfSe analysis of cervicovaginal microbiota in different cervical lesions in relation to vaginal microenvironment status. The horizontal axis shows the LDA score, indicating the influence of various bacterial genera. No significant impact of cervicovaginal microbiota was observed in the NC and CIN groups in relation to vaginal cleanness status and in the SCC group in relation to vaginal pH, H_2_O_2_, and cleanness status, so the histogram could not be generated.

*Lactobacillus* remained dominant in the NC group with a normal vaginal microenvironment. However, in the participants with an abnormal vaginal microenvironment, *Lactobacillus* proportion decreased with increasing severity of cervical lesions, whereas anaerobe levels increased (Fig. 2A). CVM diversity under an abnormal vaginal microenvironment was higher at each stage of cervical lesions, especially in the CIN patients with abnormal vaginal pH and H_2_O_2_ (Fig. 2B). *Lactobacillus* abundance was significantly higher in the NC and CIN groups with normal vaginal pH and H_2_O_2_, whereas *Aerococcus*, *Anaerococcus*, *Dialister*, and *Gardnerella* were over-represented in the NC or CIN participants with abnormal vaginal pH and H_2_O_2_. No significant characteristic bacteria were found in the NC group and CIN group depending on the cleanness status, and no significant variations in characteristic bacteria were observed in the SCC group under different microenvironmental conditions (Fig. 2C).

The GMDR model explored the interaction effect between CVM and the vaginal microenvironment in cervical carcinogenesis. CVM diversity was classified as high (>0.81) and low (≤0.81) according to the mean value of the Shannon index (0.81) in the NC group. Compared with the NC group, a significant interaction effect among abnormal vaginal H_2_O_2_, vaginal cleanness, and high CVM diversity was found in the CIN group. Similarly, compared with the NC group, a significant interaction effect was found between abnormal vaginal pH, NLD, and high CVM diversity in the SCC group (Table 3).

**TABLE 3:** Interaction of cervicovaginal microbiota combined with microenvironment factors on cervical carcinogenesis by GMDR analysis^*b*^^,^^*c*^

Groups	Model	TBA	CVC	*P* value
CIN	A2	0.658	8/10	7 (0.172)
	A2/A5^*a*^	0.706	10/10	10 (0.001)
	A2/A3/A5	0.715	5/10	9 (0.011)
SCC	A4	0.863	8/10	4 (0.828)
	A4/A5	0.960	9/10	9 (0.011)
	A1/A4/A5^*a*^	0.997	10/10	10 (0.001)

^*a*^ The best interaction model was adjusted for age, passive smoking, frequency of bathing, frequency of pubic cleaning, frequency of washing underwear, history of vaginitis, age at the first intercourse, parity, menopause, and HPV infection type.

^*b*^ CVC, cross-validation consistency; GMDR, generalized multifactor dimensionality reduction; TBA, testing balance accuracy.

^*c*^ A1–A5 represent vaginal pH, vaginal H_2_O_2_, vaginal cleanness, *Lactobacillus* dominance, and cervicovaginal microbiota diversity, respectively. Here, the cervicovaginal microbiota diversity was classified as high (>0.81) and low (≤0.81) according to the mean value of the Shannon index (0.81) in the NC group.

### Co-occurrence network and function of CVM combined with microenvironment change in different stages of cervical lesions

**Fig 3:**
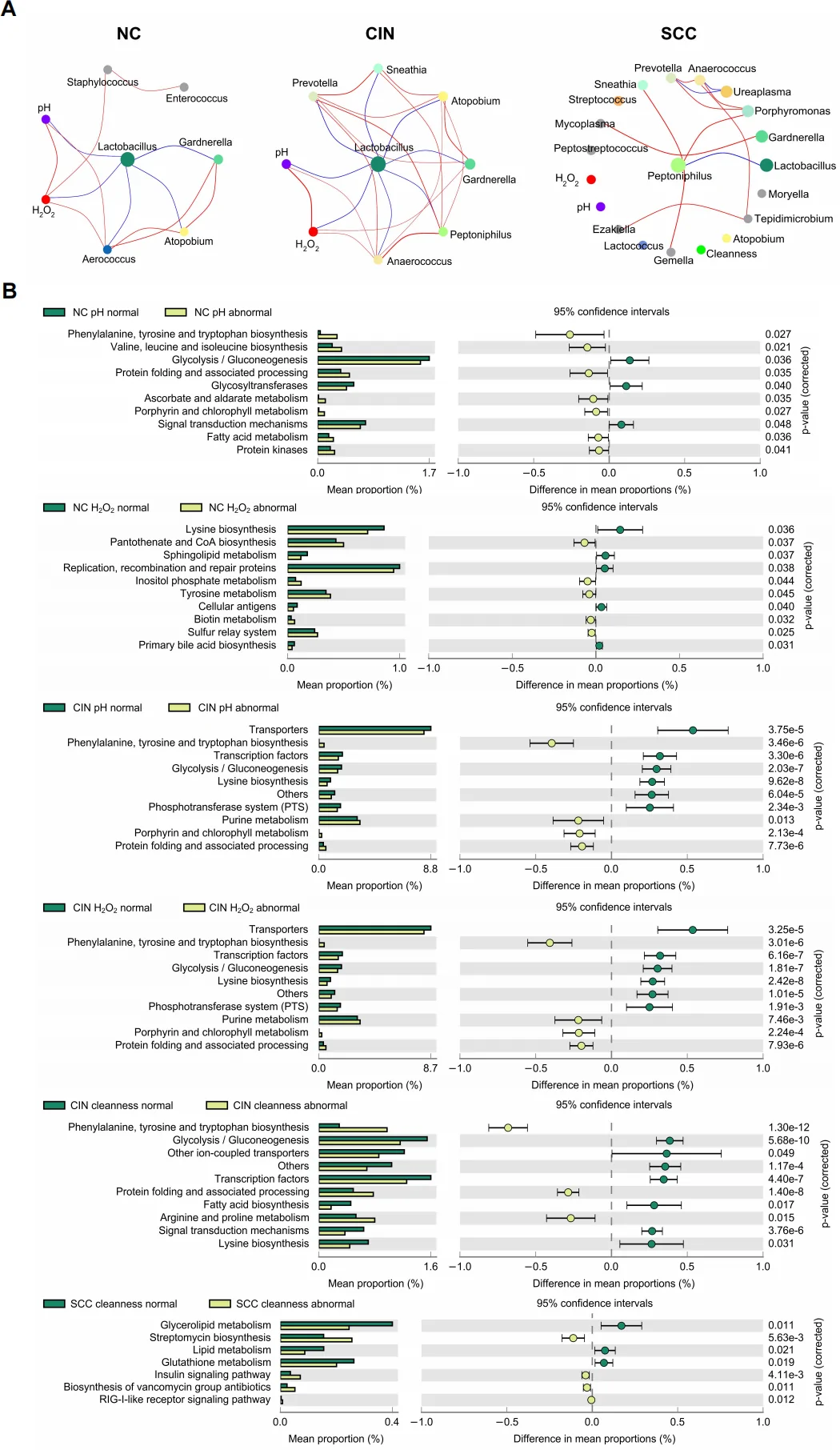
Co-occurrence network and function of cervicovaginal microbiota combined with vaginal microenvironment in cervical carcinogenesis. (**A**) Co-occurrence network of cervicovaginal microbiota combined with vaginal microenvironment factors across different stages of cervical lesions. The nodes in the figure represent different genera or factors. A positive correlation is presented as a red line, and a negative correlation is shown as a blue line, according to the Spearman’s correlation coefficient (*ρ*) > 0.3 and *P* < 0.05 by the Spearman’s rank correlation test. (**B**) Cervicovaginal microbial functions under different vaginal microenvironments in cervical carcinogenesis, showing the main function (mean proportion, %) of CVM under different normal or abnormal vaginal microenvironments (left figure) and the corresponding 95% CI (right figure). *P* < 0.05 was considered significant.

In the NC and CIN groups, *Lactobacillus* was central in the co-occurrence network and negatively correlated with abnormal vaginal pH and H_2_O_2_, whereas *Peptoniphilus* was central in the SCC group. Notably, in the CIN group, *Anaerococcus* formed a positively co-occurring relationship with abnormal vaginal pH, H_2_O_2_, and other anaerobes, such as *Gardnerella*, *Atopobium*, *Peptoniphilus*, and *Prevotella*. Similar results were not observed in the SCC group (Fig. 3A). CVM functions varied significantly with changes in the vaginal microenvironment across different stages in cervical lesions (Fig. 3B). CVM dysbiosis alters cervicovaginal metabolic profiles, creating an environment conducive to HPV persistence and cervical carcinogenesis, with the composition of CVM, particularly the dominance of specific *Lactobacillus* species, significantly influencing HPV infection and persistence (17).

## DISCUSSION

Several studies have highlighted the role of CVM and vaginal microenvironment in HPV infection in the progression of cervical carcinogenesis (18–21). Our findings support these studies, demonstrating that high CVM diversity, non-*Lactobacillus* dominance, and overgrowth of anaerobes, especially *Peptoniphilus*, *Porphyromonas*, *Anaerococcus*, *Prevotella*, *Sneathia*, and *Streptococcus*, were closely linked to the increasing severity of cervical lesions. Anaerobes are associated with persistent HPV infection and cervical dysplasia progression (22). Wu et al. (23) found that cervical microbiota diversity was higher in cases of severe cervical pathology. These cases were characterized by a lower relative abundance of *Lactobacillus* and a higher abundance of anaerobic bacteria, such as *Porphyromonas*, *Prevotella*, and *Sneathia*. Additionally, *Gardnerella* and *Atopobium* enrichment has been linked to a higher risk of CIN (24). In our study, *Gardnerella* and *Atopobium* were significantly enriched in the CIN group, indicating their potential as biomarkers for CIN. These findings underscore that CVM dysbiosis, particularly the decrease in *Lactobacillus* and the increase in anaerobes, is crucial in promoting the malignant progression of cervical lesions.

The relationship between vaginal microbiota disorders and the vaginal microenvironment is noteworthy. Our previous work showed that elevated vaginal pH, decreased H_2_O_2_, and abnormal vaginal cleanness increased the risk for cervical lesions (25). The present study revealed a significant rising trend of abnormal vaginal pH and H_2_O_2_ with the progression of cervical carcinogenesis. The deteriorated vaginal microenvironment exacerbated the decline of *Lactobacillus* and the growth of anaerobes such as *Gardnerella*, *Prevotella*, and *Peptoniphilus*. The majority of the identified anaerobes, such as *Aerococcus*, *Anaerococcus*, *Dialister*, and *Gardnerella*, were more enriched in NC or CIN women with abnormal vaginal pH or H_2_O_2_ (26). Anaerobes can form a dense biofilm that resists H_2_O_2_ and increases vaginal pH by producing amines, which promote the growth of inflammation-related microorganisms and may cause cervical lesions (27). Our results suggest a novel interaction effect between abnormal vaginal pH, non-*Lactobacillus* dominance, and increased CVM diversity in SCC, indicating that the combination of CVM dysbiosis and microenvironment disorder is closely tied to cervical carcinogenesis.

We further explored the co-occurrence network of CVM combined with the microenvironment profile in different stages of cervical lesions. This approach illustrates the relationship between the most abundant or core microorganisms and correlates them with environmental key factors (28). Wei et al. (29) depicted the vaginal microbial co-occurrence network in women with HPV infection, showing that several anaerobes negatively correlated with *Lactobacillus*. In our study, we found positive correlations among *Anaerococcus*, abnormal vaginal pH and H_2_O_2_, and other anaerobes in the CIN group. *Peptoniphilus* was central in the SCC group’s co-occurrence network, especially under abnormal vaginal pH and H_2_O_2_ conditions. *Anaerococcus*, which produces alkaline enzymes but not catalase (30), is associated with CIN progression (31). *Peptoniphilus*, producing butyric acid, could induce latent virus activation by inhibiting histone deacetylases (32). These findings suggest that increased *Peptoniphilus* might be a vital sign of cervical carcinogenesis, particularly under an abnormal vaginal microecological status.

The KEGG pathway enrichment analysis assessed CVM functions under different vaginal microenvironment states across the stages of cervical lesions. We found that the NC and CIN women with abnormal vaginal microenvironment had higher levels of phenylalanine, tyrosine, and tryptophan biosynthesis, whose metabolites provide necessary nitrogen to cancer cells and could induce inflammation (33). Our results also revealed significantly decreased metabolic functions of glycerolipid, lipid, and glutathione in the SCC patients with abnormal vaginal cleanness. This suggests that an altered metabolism pathway in CVM, along with a deteriorated microenvironment, might promote cervical carcinogenesis. Glycerolipid metabolism has been associated with infection by BV-related microorganisms (11), and its metabolites are crucial for epithelial barrier function (34). Glutathione, a key antioxidant, exhibits antiproliferative, antiapoptotic, and anti-inflammatory effects (35). Reduced glutathione is associated with HPV and may represent increased oxidative stress, which can compromise normal host responses to HPV infection and elevate the risk of cervical lesion progression.

To our knowledge, this is the first study to evaluate the influence and function of CVM combined with vaginal microenvironment profiles and explore their interaction and co-occurring relationship in cervical carcinogenesis. Our findings highlight a novel biological function of CVM under the vaginal microenvironment and suggest potential therapeutic applications for cervical cancer. This study offers a new perspective on preventing and controlling the malignant transformation of cervical lesions by maintaining CVM balance and improving the vaginal microenvironment. However, given the cross-sectional design, this study lacks the ability to prove a causal relationship between the changes in cervicovaginal microbiota (CVM) and cervical lesion progression. In addition, although 16S sequencing can provide preliminary insights into vaginal flora at the genus level, it misses much of the microbial information beyond the 16S gene. Furthermore, the small sample size is another limitation of this study, which may affect the generalizability and statistical power of the findings. Therefore, the next study will involve following up with the research participants and using metagenomic research methods to further explore the causal relationship between the changes in CVM and cervical lesion progression.

In conclusion, CVM dysbiosis and its associated functional changes, especially when combined with an abnormal vaginal microenvironment, contribute to cervical carcinogenesis and could serve as valuable predictors for developing cervical dysplasia and progressing to invasive carcinoma. Our findings provide evidence that complex vaginal microenvironment-microbe interactions are hallmarks of cervical carcinogenesis and could be valuable targets for future diagnosis, prevention, and therapeutic interventions.

## Supplementary Material

Reviewer comments

## Data Availability

Raw data were generated at the Department of Epidemiology, Shanxi Medical University. Derived data supporting the findings of this study are available from the corresponding author, Jintao Wang, upon request.

## AFTER EPUB

*[This article was published on 16 April 2026 with an error in the affiliations. The affiliations were corrected in the current version, posted on 18 June 2026.]*

## SUPPLEMENTAL MATERIAL

Figure S1 spectrum.02804-24-s0001.tif Differences in the top 9 cervicovaginal microbiota abundance of different cervical cancerization groups.

Supplemental legend spectrum.02804-24-s0002.docx Descriptive legend for Fig. S1.

Table S1 spectrum.02804-24-s0003.xlsx Alpha diversity of the cervicovaginal microbiota data.

OPEN PEER REVIEW reviewer-comments.pdf An accounting of the reviewer comments and feedback.

ASM does not own the copyrights to Supplemental Material that may be linked to, or accessed through, an article. The authors have granted ASM a non-exclusive, world-wide license to publish the Supplemental Material files. Please contact the corresponding author directly for reuse.
