# Supplementary figures and images for: Cervicovaginal dysbiosis and microenvironment disruption are associated with cervical carcinogenesis
Source: Microbiol Spectr. 2026 Apr 16;14(7):e02804-24. doi: 10.1128/spectrum.02804-24 (PMC13340114; doi:10.1128/spectrum.02804-24)

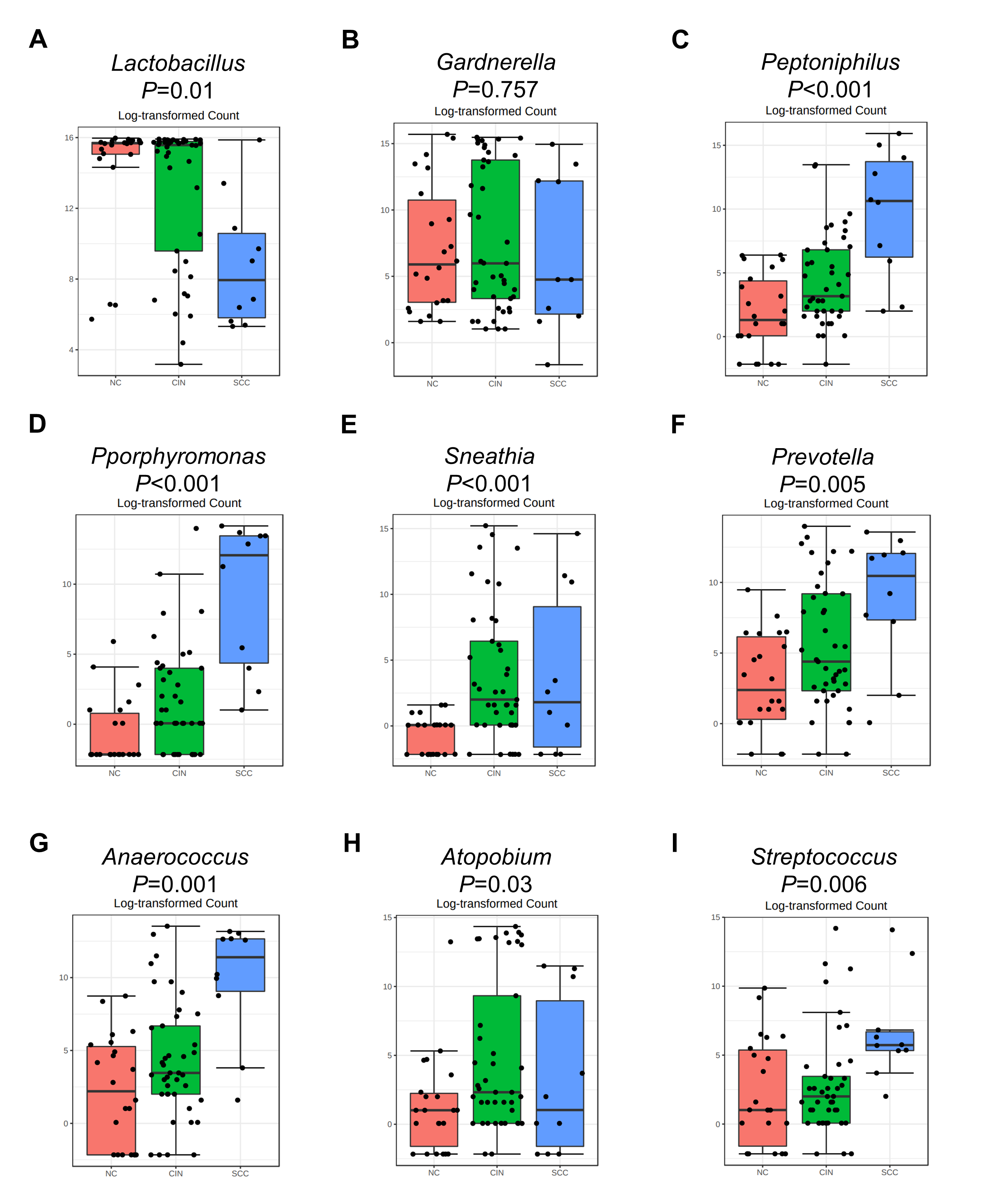

Supplement: Figure S1 — Differences in the top 9 cervicovaginal microbiota abundance of different cervical cancerization groups. [file spectrum.02804-24-s0001.tif]
