# Supplementary material for: Cervicovaginal dysbiosis and microenvironment disruption are associated with cervical carcinogenesis
Source: Microbiol Spectr. 2026 Apr 16;14(7):e02804-24. doi: 10.1128/spectrum.02804-24 (PMC13340114; doi:10.1128/spectrum.02804-24)
Supplement: Supplemental legend — Descriptive legend for Fig. S1. [file spectrum.02804-24-s0002.docx]

**Supplemental Figure 1. Differences in the top9 cervicovaginal microbiota abundance of different cervical cancerization groups**

Log-transformation of the abundance of cervicovaginal microbiota was performed. P-values were calculated by Kruskal-Wallis test. P<0.05 means significant.
