# Supplementary material for: Cervicovaginal dysbiosis and microenvironment disruption are associated with cervical carcinogenesis
Source: Microbiol Spectr. 2026 Apr 16;14(7):e02804-24. doi: 10.1128/spectrum.02804-24 (PMC13340114; doi:10.1128/spectrum.02804-24)
Supplement: Reviewer comments [file reviewer-comments.pdf]

AMERICAN SOCIETY FOR MICROBIOLOGY | Microbiology Spectrum

Cervicovaginal dysbiosis and microenvironment disruption are associated with cervical carcinogenesis

Meng Cui, Mingxuan Zhang, Jianjun Zhang, Rui Mao, Ling Ding, Zhilian Wang, Li Song, Yuanjing Lyu, Huimin Li, Kailu Zhao, and Jintao Wang

Corresponding Author(s): Jintao Wang, Shanxi Medical University

Review Timeline:

Submission Date:	November 9, 2024
Editorial Decision:	June 25, 2025
Revision Received:	August 23, 2025
Editorial Decision:	November 24, 2025
Revision Received:	January 6, 2026
Accepted:	March 17, 2026

Editor: Jian Li

Reviewer(s): Disclosure of reviewer identity is with reference to reviewer comments included in decision letter(s). The following individuals involved in review of your submission have agreed to reveal their identity: Ashish Kothari (Reviewer #1); Rachael Terumbur Duche (Reviewer #5)

Transaction Report:

(Note: With the exception of the correction of typographical or spelling errors that could be a source of ambiguity, letters and reports are not edited. The original formatting of letters and referee reports may not be reflected in this compilation.)

DOI: https://doi.org/10.1128/spectrum.02804-24

Re: Spectrum02804-24 (**Dysbiosis of Cervicovaginal microbiota combining with microenvironment disorder promotes the adverse development of cervical cancerization**)

Dear Prof. Jintao Wang:

Thank you for the privilege of reviewing your work. Below you will find my comments, instructions from the Spectrum editorial office, and the reviewer comments.

Please return the manuscript within 60 days; if you cannot complete the modification within this time period, please contact me. If you do not wish to modify the manuscript and prefer to submit it to another journal, notify me immediately so that the manuscript may be formally withdrawn from consideration by Spectrum.

Revision Guidelines
To submit your modified manuscript, log into the submission site at https://spectrum.msubmit.net/cgi-bin/main.plex. Go to Author Tasks and click the appropriate manuscript title to begin. The information you entered when you first submitted the paper will be displayed; update this as necessary. Note the following requirements:

• Upload point-by-point responses to the issues raised by the reviewers in a file named "Response to Reviewers," NOT in your cover letter.
• Upload a compare copy of the manuscript (without figures) as a "Marked-Up Manuscript" file.
• Upload a clean .DOC/.DOCX version of the revised manuscript and remove the previous version.
• Each figure must be uploaded as a separate, editable, high-resolution file (TIFF or EPS preferred), and any multipanel figures must be assembled into one file.
• Any supplemental material intended for posting by ASM should be uploaded with their legends separate from the main manuscript. You can combine all supplemental material into one file (preferred) or split it into a maximum of 10 files with all associated legends included.

For complete guidelines on revision requirements, see our Submission and Review Process webpage. Submission of a paper that does not conform to guidelines may delay acceptance of your manuscript.

Data availability: ASM policy requires that data be available to the public upon online posting of the article, so please verify all links to sequence records, if present, and make sure that each number retrieves the full record of the data. If a new accession number is not linked or a link is broken, provide Spectrum production staff with the correct URL for the record. If the accession numbers for new data are not publicly accessible before the expected online posting of the article, publication may be delayed; please contact production staff (Spectrum@asmusa.org) immediately with the expected release date.

Publication Fees: For information on publication fees and which article types are subject to charges, visit our website. If your manuscript is accepted for publication and any fees apply, you will be contacted separately about payment during the production process; please follow the instructions in that e-mail. Arrangements for payment must be made before your article is published.

ASM Membership: Corresponding authors may join or renew ASM membership to obtain discounts on publication fees. Need to upgrade your membership level? Please contact Customer Service at Service@asmusa.org.

The ASM Journals program strives for constant improvement in our submission and publication process. Please tell us how we can improve your experience by taking this quick Author Survey.

Thank you for submitting your paper to Spectrum.

Sincerely,
Jian Li
Editor
Microbiology Spectrum

Reviewer #1 (Comments for the Author):

Overall, the authors provide a reasonably detailed and thorough characterization of the importance of addressing vaginal microecology to warn against and prevent cervical cancerization. I do believe that the present manuscript is suitable for publication in this journal. I would suggest that the authors consider the following points as they revise their manuscript and continue their work in this important research area.

1. The title accurately reflects the core findings but could be streamlined for clarity.
2. Miner grammatical issues 30-31 "while anaerobe levels increased... while the proportions of anaerobes increased"
3. The objectives of this study are absent. After the introduction, one paragraph for the objectives statement is necessary.
4. Introduction needs minor revision. The author could check the recently published studies.
5. Line 70 is not fully understood" is vague. Consider specifying which aspects remain unclear.
6. Clarify how sample sizes (n=73 for sequencing vs. n=510 total) were determined and whether power calculations were performed.
7. Please define the cut-off for high diversity (Shannon index >0.81). Justify this threshold biologically or statistically.
8. Figures 1 and 2 are statistically independent from overlapping data, please add the P values.
9. Please ensure all figures have legends that are sufficiently explanatory without referencing the main text.
10. The discussions are poorly referenced with literature that does not support the statements made in the text.
11. Kindly add one paragraph of "future aspect"
12. Numerous grammatical errors and awkward detract please consider professional English editing.
13. The limitations section should contain more prominent notes.

Reviewer #2 (Comments for the Author):

Specific comments:
• The study design is observational and cross-sectional, which limits the ability to establish causal relationships between CVM changes and cervical lesion progression for many resaons:
1- The study failed to determnie the temporal sequence for the CVM (i.e. which factor preceeds the second). Do you think that the microbiota changes is the reaseon behind the lesion development or the opposite.
2- It lacks follow-up data to determine if there is additional unknown factor affecting the microenvironement such as sexual behavior, hormonal influences, antibiotic use, or co-infections (e.g., with Chlamydia or Trichomonas) that were not explicitly controlled, which may influence both microbiota composition and cervical health.
3- If 16S rRNA gene sequencing was used, it provides limited resolution at the species level and does not reveal microbial functional capacity as precisely as metagenomic or metabolomic approaches.
4- Vaginal pH and H_2O_2 are useful indicators but can be influenced by external factors (e.g., menstruation, hygiene practices), limiting their precision in reflecting true microenvironmental status.
5- Pathways like histone deacetylase inhibition or glutathione depletion are proposed based on KEGG enrichment and existing literature, not directly measured in patient tissues.
6- While the study discusses persistent HPV infection, it does not provide direct data on HPV types, viral loads, or integration status, which are key variables in cervical carcinogenesis.
So, it is highly recommended to add a paragraph addressing the main limitations of the current study prior to the Conclusion section.
- For Discussion, it is suggested to be brief in a more concise way, to focus on the study findings, and to avoid repeated statement of the study rationale, which has been mentioned in the Materials and Methods.
- English proof reading is highly recommended.

Minor comments:
L44: For clarity, please define the HPV abbreviation before the introduction.
L59: Before mentioning lactobacillus, please provide a connective sentence.
L186: based>bsed
L260: For several studies, you included one reference; please add more
Figure 2B; why didn't you analyze the data for NC/SCC?
Please remove reference 11 and replace it with a peer-reviewed article. It is not appropriate to reference an article in "the conversation", when there are peer-reviewed journal articles that talk about the vaginal microenvironment.

Overall, the authors provide a reasonably detailed and thorough characterization of the importance of addressing vaginal microecology to warn against and prevent cervical cancerization. I do believe that the present manuscript is suitable for publication in this journal. I would suggest that the authors consider the following points as they revise their manuscript and continue their work in this important research area.

1. The title accurately reflects the core findings but could be streamlined for clarity.
2. Miner grammatical issues 30–31 “while anaerobe levels increased... while the proportions of anaerobes increased”
3. The objectives of this study are absent. After the introduction, one paragraph for the objectives statement is necessary.
4. Introduction needs minor revision. The author could check the recently published studies.
5. Line 70 is not fully understood" is vague. Consider specifying which aspects remain unclear.
6. Clarify how sample sizes (n=73 for sequencing vs. n=510 total) were determined and whether power calculations were performed.
7. Please define the cut-off for high diversity (Shannon index >0.81). Justify this threshold biologically or statistically.
8. Figures 1 and 2 are statistically independent from overlapping data, please add the P values.
9. Please ensure all figures have legends that are sufficiently explanatory without referencing the main text.
10. The discussions are poorly referenced with literature that does not support the statements made in the text.
11. Kindly add one paragraph of “future aspect”
12. Numerous grammatical errors and awkward detract please consider professional English editing.
13. The limitations section should contain more prominent notes.

Dear Editor,

Thank you very much for your kind letter and the comments concerning our manuscript entitled "**Dysbiosis of Cervicovaginal microbiota combining with microenvironment disorder promotes the adverse development of cervical cancerization**".

We greatly appreciate the comments and careful review. All of the comments provided were extremely valuable and very helpful for revising our manuscript and improving our research. We have revised the manuscript based on these comments and suggestions, and have kept the revision marks in a file titled "Marked Up Manuscript File". We hope the new manuscript will meet the magazine's standards. Below you will find our point-by-point responses to the reviewers' comments/questions.

Thank you for your continued consideration and efforts for our manuscript.

Once again, thank you and all the reviewers for the kind advice.

Should you have any questions, please contact us without hesitate.

Sincerely,

Meng Cui, Ming-xuan Zhang and Jin-tao Wang

Response to Reviewers

Reviewer #1 (Comments for the Author):

Q1. The title accurately reflects the core findings but could be streamlined for clarity.

A1：Thanks very much for your kind guidance. We revised the title to “**Cervicovaginal Dysbiosis and Microenvironment Disruption Promote Cervical Carcinogenesis”.**

Q2. Miner grammatical issues 30-31 "while anaerobe levels increased... while the proportions of anaerobes increased"

A2: Thanks very much for your kind guidance. We have already revised this sentence; the modified part is in lines 30–33. “As cervical cancerization progressed, anaerobe levels increased in the deteriorating vaginal microenvironment. Abundance of Lactobacillus gradually decreased, whereas the proportion of anaerobes increased in the deteriorating vaginal microenvironment.”

Q3. The objectives of this study are absent. After the introduction, one paragraph for the objectives statement is

necessary.

A3: Thank you very much for your kind guidance. We have added a paragraph to the introduction (line 91–93). “Herein, we aimed to highlight the role of CVM and vaginal microenvironment in HPV infection in the progression of cervical carcinogenesis. Our results may provide new insights into the etiology and pathogenesis of cervical cancer from the perspective of vaginal microecology.”

Q4. Introduction needs minor revision. The author could check the recently published studies.

A4: Thanks very much for your kind guidance. We have updated the references.

Q5. Line 70 is not fully understood" is vague. Consider specifying which aspects remain unclear.

A5: Thanks very much for your kind guidance. We add one paragraph after line 78: “Specifically, it is still unknown whether CVM dysbiosis and its associated functional changes, particularly when combined with an abnormal vaginal microenvironment, contribute to cervical carcinogenesis and could serve as valuable predictors for the development of

cervical dysplasia and the progression to invasive carcinoma."

Q6. Clarify how sample sizes (n=73 for sequencing vs. n=510 total) were determined and whether power calculations were performed.

A6: Sample size determination was based on power calculations performed prior to study initiation. For the total sample (n=510), we calculated the required sample size using $n = \frac{\left(\frac{Z_\alpha}{\delta}\right)^2 * p * (1-p)}{1 + [\left(\frac{Z_\alpha}{\delta}\right)^2 * p * (1-p)]/N}$ based on incidence of 0.019, α=0.05, power=80%, and accounting for an estimated 5% invalid questionnaire. For the sequencing subset (n=73), the sample size was determined based on the exclusion of individuals who had used antibiotics, vaginal medication or vaginal douches within three days prior to the study. These individuals were also matched for age and residence (same city/county) across different groups and selected for 16S rRNA gene sequencing.

Q7. Please define the cut-off for high diversity (Shannon index >0.81). Justify this threshold biologically or statistically.

A7: As mentioned earlier, threshold 0.81 is the mean value of the Shannon index in the normal control (NC) group (line 241-243).

Q8. Figures 1 and 2 are statistically independent from overlapping data, please add the P values.

A8：Thanks very much for your kind guidance. We added *P* values in Figure 1 D, E and Figure 2B. The Figure 1C and the Figure 2 C are LDA scores, which has a positive correlation with diversity of cervicovaginal microbiota. *, showed a statistical significance difference (*P*<0.05).

Q9. Please ensure all figures have legends that are sufficiently explanatory without referencing the main text.

A9: Thanks very much for your kind guidance. We added legends to figures.

Q10. The discussions are poorly referenced with literature that does not support the statements made in the text.

A10: Thanks very much for your kind guidance. We have revised and updated the literature under discussion in revised manuscript.

Q11. Kindly add one paragraph of "future aspect"

A11: Thanks very much for your kind guidance. We have

added one paragraph of future aspect (line 331-333). “Therefore, the next study will involve following up with the research participants and using metagenomic research methods to further explore the causal relationship between the changes in CVM and cervical lesion progression.”

Q12. Numerous grammatical errors and awkward detract please consider professional English editing.

A12: Thank you very much for valuable advice. We have enhanced the clarity and readability of English language by LetPub (www.letpub.com.cn) editing service.

Q13. The limitations section should contain more prominent notes.

A13: Thank you very much for valuable advice. We have improved limitations section (line 328-331). “However, given the cross-sectional design, this study lacks the ability to prove a causal relationship between the changes in CVM and cervical lesion progression. In addition, although 16S sequencing can provide preliminary insights into vaginal flora at the genus level, it misses much of the microbial information beyond the 16S gene.”

Reviewer #2 (Comments for the Author):

• The study design is observational and cross-sectional, which limits the ability to establish causal relationships between CVM changes and cervical lesion progression for many resaons:

Q1：The study failed to determnie the temporal sequence for the CVM (i.e. which factor preceeds the second). Do you think that the microbiota changes is the reaseon behind the lesion development or the opposite.

A1: Thank you for your advice. We believe that vaginal flora and microenvironmental factors play a role in the occurrence and development of cervical lesions. This is one of the causes of cervical lesions. Notably, serial follow-up data showed that these microbial alterations were present for at least one to two years and occurred before pathological changes. [Cervicovaginal microbiota long-term dynamics and prediction of different outcomes in persistent human papillomavirus infection]，[Temporal composition of the cervicovaginal microbiome associates with hrHPV infection outcomes in a longitudinal study]

Q2: It lacks follow-up data to determine if there is additional unknown factor affecting the microenvironement such as sexual behavior, hormonal influences, antibiotic use, or co-infections (e.g., with Chlamydia or Trichomonas) that

were not explicitly controlled, which may influence both microbiota composition and cervical health.

A2: To prevent interference from other unknown factors, 73 women who had not used antibiotics or anti-inflammatory drugs within the last two weeks, had not had sexual intercourse within the last three days, and had not undergone vaginal douching within the last 48 hours were selected for sequencing. These cases were analysed to determine whether the women had experienced vaginitis (including Chlamydia or Trichomonas co-infection) in the past. No statistically significant differences were found. Information on sexual behaviour is analysed in Table 1.

Q3: If 16S rRNA gene sequencing was used, it provides limited resolution at the species level and does not reveal microbial functional capacity as precisely as metagenomic or metabolomic approaches.

A3: Preliminary exploration of vaginal flora at the genus level using 16S rRNA indeed missed a large amount of microbial information beyond the 16S gene and is therefore limited. However, our team later conducted related metagenomics research.

Q4: Vaginal pH and H_2O_2 are useful indicators but can be influenced by external factors (e.g., menstruation, hygiene practices), limiting their precision in reflecting true microenvironmental status.

A4: We added the results of the analysis of related factors such as menstruation and hygiene habits in Table 1

Q5: Pathways like histone deacetylase inhibition or glutathione depletion are proposed based on KEGG enrichment and existing literature, not directly measured in patient tissues.

A5: This study is a preliminary exploration based on KEGG, and further analysis will be considered in the future.

Q6: While the study discusses persistent HPV infection, it does not provide direct data on HPV types, viral loads, or integration status, which are key variables in cervical carcinogenesis.

A6: Thank you for your advice. We have included data on HPV types in the analysis in Table 1.

Q7: So, it is highly recommended to add a paragraph addressing the main limitations of the current study prior to the Conclusion section.

A7: Thank you for your advice. We have added a paragraph addressing the main limitations of the current study prior to the conclusion section (line 328-333).

Minor comments:

L44: For clarity, please define the HPV abbreviation before the introduction.

Answer: The modified position is line 46.

L59: Before mentioning lactobacillus, please provide a connective sentence.

Answer: Added in line 64-66.

L186: based> bsed

Answer: This sentence has been modified (line 200-202).

L260: For several studies, you included one reference; please add more

Answer: Added in line 270 in revised manuscript.

Figure 2B; why didn't you analyze the data for NC/SCC?

Answer: The data are not marked with * because they are not statistically significant, or because the sample size is too small (see the red groups in the figure). Therefore, inter-group comparison is not possible.

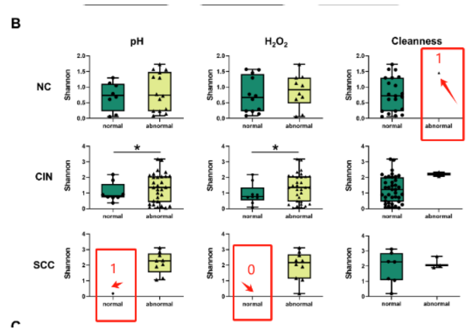

Please remove reference 11 and replace it with a peer-reviewed article. It is not appropriate to reference an article in "the conversation", when there are peer-reviewed journal articles that talk about the vaginal microenvironment.

Answer: Thank you for your advice, we have replaced the reference 11.

Answer for Minor comments:

Thank you for your advice. We have revised the above question according to your suggestion.

Re: Spectrum02804-24R1 (**Cervicovaginal dysbiosis and microenvironment disruption promote cervical carcinogenesis**)

Dear Prof. Jintao Wang:

Thank you for the privilege of reviewing your work. Below you will find my comments, instructions from the Spectrum editorial office, and the reviewer comments.

Please return the manuscript within 60 days; if you cannot complete the modification within this time period, please contact me. If you do not wish to modify the manuscript and prefer to submit it to another journal, notify me immediately so that the manuscript may be formally withdrawn from consideration by Spectrum.

Revision Guidelines
To submit your modified manuscript, log into the submission site at https://spectrum.msubmit.net/cgi-bin/main.plex. Go to Author Tasks and click the appropriate manuscript title to begin. The information you entered when you first submitted the paper will be displayed; update this as necessary. Note the following requirements:

• Upload point-by-point responses to the issues raised by the reviewers in a file named "Response to Reviewers," NOT in your cover letter.
• Upload a compare copy of the manuscript (without figures) as a "Marked-Up Manuscript" file.
• Upload a clean .DOC/.DOCX version of the revised manuscript and remove the previous version.
• Each figure must be uploaded as a separate, editable, high-resolution file (TIFF or EPS preferred), and any multipanel figures must be assembled into one file.
• Any supplemental material intended for posting by ASM should be uploaded with their legends separate from the main manuscript. You can combine all supplemental material into one file (preferred) or split it into a maximum of 10 files with all associated legends included.

For complete guidelines on revision requirements, see our Submission and Review Process webpage. Submission of a paper that does not conform to guidelines may delay acceptance of your manuscript.

Data availability: ASM policy requires that data be available to the public upon online posting of the article, so please verify all links to sequence records, if present, and make sure that each number retrieves the full record of the data. If a new accession number is not linked or a link is broken, provide Spectrum production staff with the correct URL for the record. If the accession numbers for new data are not publicly accessible before the expected online posting of the article, publication may be delayed; please contact production staff (Spectrum@asmusa.org) immediately with the expected release date.

Publication Fees: For information on publication fees and which article types are subject to charges, visit our website. If your manuscript is accepted for publication and any fees apply, you will be contacted separately about payment during the production process; please follow the instructions in that e-mail. Arrangements for payment must be made before your article is published.

ASM Membership: Corresponding authors may join or renew ASM membership to obtain discounts on publication fees. Need to upgrade your membership level? Please contact Customer Service at Service@asmusa.org.

The ASM Journals program strives for constant improvement in our submission and publication process. Please tell us how we can improve your experience by taking this quick Author Survey.

Thank you for submitting your paper to Spectrum.

Sincerely,
Jian Li
Editor
Microbiology Spectrum

Reviewer #1 (Comments for the Author):

this version is definitely suitable for publication.

Reviewer #4 (Comments for the Author):

1. The author should introduce the the specific pathways after this sentence-"CVM functions varied significantly with changes in the vaginal microenvironment across different stages of cervical lesions"
2. L33, Lactobacillus should be in italics
3. The small sample size used for sequencing is also a limitation of this study
4. The research content does not match the title of the manuscript. This study is merely a cross-sectional study, which can not draw a causal view. Thus, the title exaggerates the main findings of this study

Reviewer #5 (Public repository details (Required)):

The alpha diversity of the cervicovaginal microbiota need to be deposited in a public repository for a follow-up research and for reference purposes.

Reviewer #5 (Comments for the Author):

The study presented a sound experimental and methodological rigor which has actually led to the achievement of the stated objective.

This study explored the relationships among cervicovaginal microbiota, vaginal microenvironment profiles and cervical carcinogenesis. Here are some comments concerning this manuscript:

1. The author should introduce the the specific pathways after this sentence-“CVM functions varied significantly with changes in the vaginal microenvironment across different stages of cervical lesions”.

2. L33, Lactobacillus should be in italics.

3. The small sample size used for sequencing is also a limitation of this study.

4. The research content does not match the title of the manuscript. This study is merely a cross-sectional study, which can not draw a causal view. Thus, the title exaggerates the main findings of this study.

Dear Editor, thank you very much for giving me the opportunity to be part of the review team for this manuscript titled: "**Cervicovaginal dysbiosis and microenvironment disruption promote cervical carcinogenesis.**" By Meng Cui and others.

I have seen that a decision letter was issued by the Editor to the authors from previous review. I have also gone through the corrective and convincing responses by authors vis-a vis. However, I still have a few observations that require only minor modifications:

1. Title is okay.
2. Abstract needs restructuring. One or two sentences will suffice for the abstract background; the aim of the work should be clearly stated in a sentence in the abstract. There is repetition in line 32-34. You may consider writing it thus: "As cervical cancerization progressed, the abundance of Lactobacillus gradually decreased with thriving anaerobe levels in the deteriorating vaginal microenvironment."
3. Introduction: Ln 76 – 80 can be broken down to two sentences. The sentence is unnecessarily long thereby losing its meaning; Ln 79, authors may consider the word "markers" for "predictors"; Ln 84, "indexes" should be "indices";
4. Materials and methods: Design is okay; Ln 111, spacing.
5. Results: Presentation is okay with accurate statistical analysis.
6. Discussion: Ln 273 – 274, "several studies"… but you mentioned only two (2); two cannot be several but a few. Overall, the discussion given by the authors is concise with extensive corroborations with other studies.

   The results obtained in this study are relevant for public health consumption.

Dear Editor,

Thank you very much for your kind letter and the comments concerning our manuscript entitled " **Dysbiosis of Cervicovaginal microbiota combining with microenvironment disorder promotes the adverse development of cervical cancerization**" .

We greatly appreciate the comments and careful review. All of the comments provided were extremely valuable and very helpful for revising our manuscript and improving our research. Based on these comments and suggestions, we have revised the manuscript and kept the revision marks. We hope the new manuscript will meet the magazine's standards. Below you will find our point-by-point responses to the reviewers' comments/questions.

Thank you for your continued consideration and efforts for our manuscript.

Once again, thank you and all the reviewers for the kind advice.

Should you have any questions, please contact us without hesitate.

Sincerely,

Meng Cui, Ming-xuan Zhang and Jin-tao Wang

Response to Reviewers

Reviewer #4 (Comments for the Author):

Q1. The author should introduce the the specific pathways after this sentence “CVM functions varied significantly with changes in the vaginal microenvironment across different stages of cervical lesions”.

A1：Thanks very much for your kind guidance. In line 234, we introduce the specific pathways of CVM functions varied significantly with changes in the vaginal microenvironment across different stages in cervical lesions.

Q2. L33, Lactobacillus should be in italics.

A2：Thanks very much for your kind guidance. We have modify the format.

Q3. The small sample size used for sequencing is also a limitation of this study

A3：Thanks very much for your kind guidance. We have added this limitation in the discussion in line 302.

Q4. The research content does not match the title of the manuscript. This study is merely a cross-sectional study,

which can not draw a causal view. Thus, the title exaggerates the main findings of this study.

A3: Thanks very much for your kind guidance. We have changed the title to "Cervicovaginal dysbiosis and microenvironment disruption are associated with cervical carcinogenesis".

Reviewer #5 (Comments for the Author):

Q1. Abstract needs restructuring. One or two sentences will suffice for the abstract background; the aim of the work should be clearly stated in a sentence in the abstract. There is repetition in line 32-34. You may consider writing it thus: "As cervical cancerization progressed, the abundance of Lactobacillus gradually decreased with thriving anaerobe levels in the deteriorating vaginal microenvironment."

A1: Thank you very much for your kind guidance. We have already revised this sentence according to your suggestion in line 32.

Q2. 3. Introduction: Ln 76 – 80 can be broken down to two sentences. The sentence is unnecessarily long thereby losing its meaning; Ln 79, authors may consider the word "markers" for "predictors"; Ln 84, "indexes" should be "indices";

A2: Thank you very much for your kind guidance. We have already revised this sentence according to your suggestion in line 75-79，83.

Q3. Materials and methods: Design is okay; Ln 111, spacing.

A3: Thank you very much for your kind guidance. We have already added this spacing in line 110.

Q4. Discussion: Ln 273 – 274, “several studies”… but you mentioned only two (2); two cannot be several but a few.

A4: Thank you very much for your kind guidance. We have already added references in line 240.

Q5. The alpha diversity of the cervicovaginal microbiota need to be deposited in a public repository for a follow-up research and for reference purposes.

A5: Thank you very much for your kind guidance. We have already upload the alpha diversity of the cervicovaginal microbiota data to the attachment.

Re: Spectrum02804-24R2 (**Cervicovaginal dysbiosis and microenvironment disruption are associated with cervical carcinogenesis**)

Dear Prof. Jintao Wang:

Your manuscript has been accepted, and I am forwarding it to the ASM production staff for publication. Your paper will first be checked to make sure all elements meet the technical requirements. ASM staff will contact you if anything needs to be revised before copyediting and production can begin. Otherwise, you will be notified when your proofs are ready to be viewed.

Data Availability: ASM policy requires that data be available to the public upon online posting of the article, so please verify all links to sequence records, if present, and make sure that each number retrieves the full record of the data. If a new accession number is not linked or a link is broken, provide production staff with the correct URL for the record. If the accession numbers for new data are not publicly accessible before the expected online posting of the article, publication may be delayed; please contact ASM production staff immediately with the expected release date.

Publication Fees: For information on publication fees and which article types have charges, please visit our website. We have partnered with Copyright Clearance Center (CCC) to collect author charges. If fees apply to your paper, you will receive a message from no-reply@copyright.com with further instructions. For questions related to paying charges through RightsLink, please contact CCC at ASM_Support@copyright.com or toll free at +1-877-622-5543. CCC makes every attempt to respond to all emails within 24 hours.

ASM Membership: Corresponding authors may join or renew ASM membership to obtain discounts on publication fees. Need to upgrade your membership level? Please contact Customer Service at Service@asmusa.org.

Embargo Policy: A press release may be issued as soon as the manuscript is posted on the Spectrum Latest Articles webpage. The corresponding author will receive an email with the subject line "ASM Journals Author Services Notification" when the article is available online.

The ASM Journals program strives for constant improvement in our submission and publication process. Please tell us how we can improve your experience by taking this quick Author Survey.

Thank you for submitting your paper to Spectrum.

Sincerely,
Jian Li
Editor
Microbiology Spectrum

Reviewer #1 (Comments for the Author):

Thanks for addressing my comments and revising the manuscript accordingly. I appreciate the author's careful responses and the improvements made in the revised version.
I have no further comments.

Reviewer #5 (Public repository details (Required)):

The Alpha Diversity of Cervicovaginal microbiota data

Reviewer #5 (Comments for the Author):

All concerns raised in the previous review have been addressed

Dear Editor, thank you very much for giving me the opportunity to be part of the review team for this manuscript titled: "**Cervicovaginal dysbiosis and microenvironment disruption promote cervical carcinogenesis."** By Meng Cui and others.

I have seen that a decision letter was issued by the Editor to the authors from previous review. I have also gone through the corrective and convincing responses by authors vis-a vis. However, I still have a few observations that require only minor modifications:

1. Title is okay.
2. Abstract needs restructuring. One or two sentences will suffice for the abstract background; the aim of the work should be clearly stated in a sentence in the abstract. There is repetition in line 32-34. You may consider writing it thus: "As cervical cancerization progressed, the abundance of Lactobacillus gradually decreased with thriving anaerobe levels in the deteriorating vaginal microenvironment."
3. Introduction: Ln 76 – 80 can be broken down to two sentences. The sentence is unnecessarily long thereby losing its meaning; Ln 79, authors may consider the word "markers" for "predictors"; Ln 84, "indexes" should be "indices";
4. Materials and methods: Design is okay; Ln 111, spacing.
5. Results: Presentation is okay with accurate statistical analysis.
6. Discussion: Ln 273 – 274, "several studies"… but you mentioned only two (2); two cannot be several but a few. Overall, the discussion given by the authors is concise with extensive corroborations with other studies. The results obtained in this study are relevant for public health consumption.
